# Supplementary material for: Novel Driver Strength Index highlights important cancer genes in TCGA PanCanAtlas patients
Source: PeerJ. 2022 Aug 11;10:e13860. doi: 10.7717/peerj.13860 (PMC9375969; doi:10.7717/peerj.13860)
Supplement: Supplemental Information 2 [file peerj-10-13860-s002.zip › COHORTS/genes plots/stage/2021_8_16_14_9_distribution_stage_III.pdf]

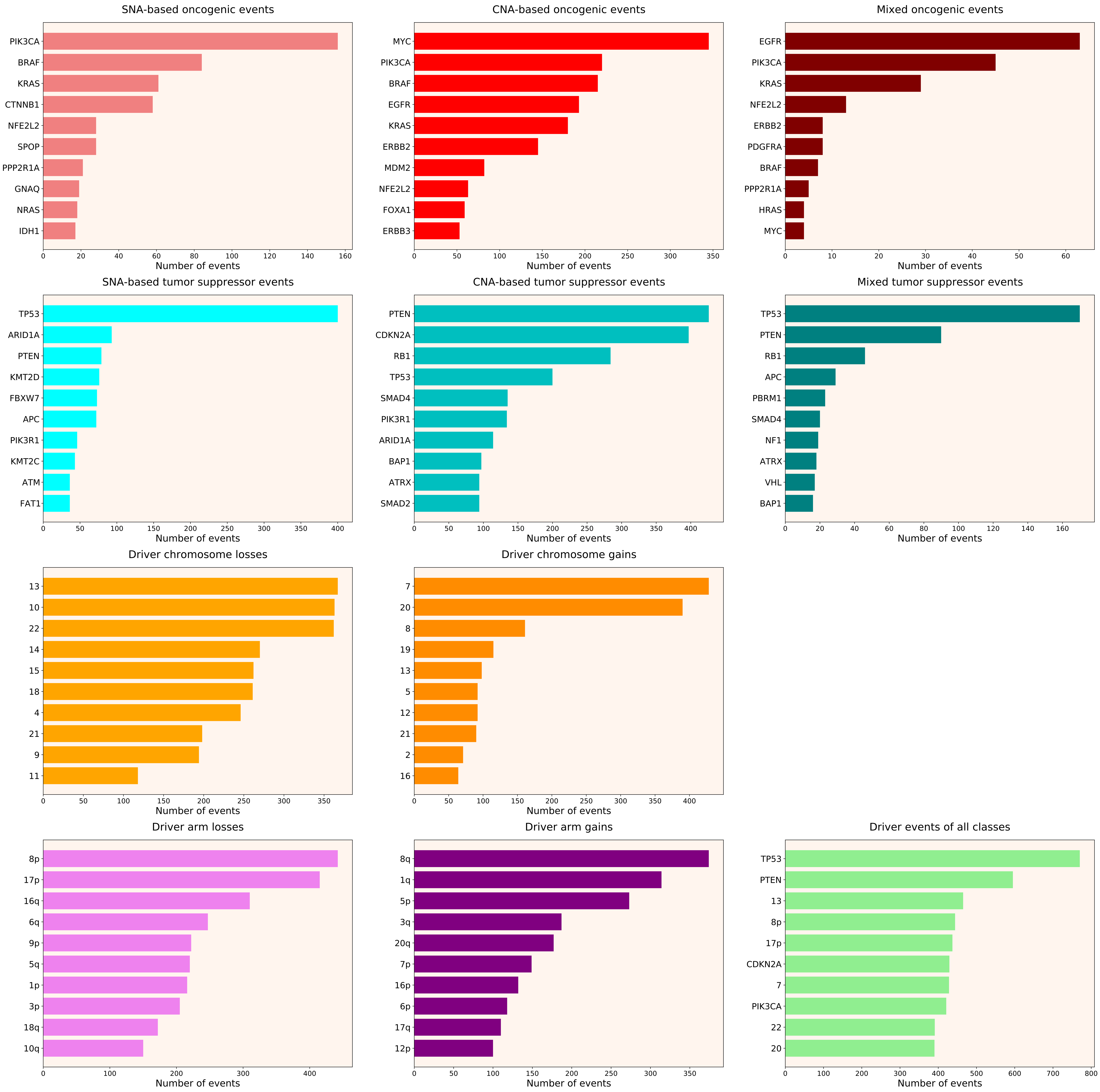
SNA-based oncogenic events
PIK3CA
BRAF
KRAS
CTNNB1
NFE2L2
SPOP
PPP2R1A
GNAQ
NRAS
IDH1
0 20 40 60 80 100 120 140 160
Number of events
CNA-based oncogenic events
MYC
PIK3CA
BRAF
EGFR
KRAS
ERBB2
MDM2
NFE2L2
FOXA1
ERBB3
0 50 100 150 200 250 300 350
Number of events
Mixed oncogenic events
EGFR
PIK3CA
KRAS
NFE2L2
ERBB2
PDGFRA
BRAF
PPP2R1A
HRAS
MYC
0 10 20 30 40 50 60
Number of events
SNA-based tumor suppressor events
TP53
ARID1A
PTEN
KMT2D
FBXW7
APC
PIK3R1
KMT2C
ATM
FAT1
0 50 100 150 200 250 300 350 400
Number of events
CNA-based tumor suppressor events
PTEN
CDKN2A
RB1
TP53
SMAD4
PIK3R1
ARID1A
BAP1
ATRX
SMAD2
0 50 100 150 200 250 300 350 400
Number of events
Mixed tumor suppressor events
TP53
PTEN
RB1
APC
PBRM1
SMAD4
NF1
ATRX
VHL
BAP1
0 20 40 60 80 100 120 140 160
Number of events
Driver chromosome losses
13
10
22
14
15
18
4
21
9
11
0 50 100 150 200 250 300 350
Number of events
Driver chromosome gains
7
20
8
19
13
5
12
21
2
16
0 50 100 150 200 250 300 350 400
Number of events
Driver arm losses
8p
17p
16q
6q
9p
5q
1p
3p
18q
10q
0 100 200 300 400
Number of events
Driver arm gains
8q
1q
5p
3q
20q
7p
16p
6p
17q
12p
0 50 100 150 200 250 300 350
Number of events
Driver events of all classes
TP53
PTEN
13
8p
17p
CDKN2A
7
PIK3CA
22
20
0 100 200 300 400 500 600 700 800
Number of events
